# Fatal *Mycobacterium avium* meningitis in an HIV-negative Vietnamese man: a case report

**DOI:** 10.1186/s13256-025-05273-4

**Published:** 2025-05-15

**Authors:** Van Thanh Nguyen, Le Hong Van, Guy Thwaites, Nguyen Thuy Thuong Thuong, Pham Kieu Nguyet Oanh, Do Dang Anh Thu, Nguyen Thanh Dung, Van Thi Xuan Quynh, Nguyen Tran Thuong Dinh, Ho Dang Trung Nghia

**Affiliations:** 1 Oxford University Clinical Research Unit, Centre for Tropical Medicine, Ho Chi Minh City, 700000 Vietnam; 2 Centre for Tropical Medicine and Global Health, Nuffield Department of Medicine, University of Oxford, Oxford, OX3 7LG UK; 3 Hospital for Tropical Diseases, Ho Chi Minh City, 700000 Vietnam; 4 Infectious Diseases Department, Pham Ngoc Thach Medical University, Ho Chi Minh City, 700000 Vietnam

**Keywords:** *Mycobacterium avium* complex, Meningitis, Non-tuberculous mycobacterial meningitis, Non-HIV, Case report

## Abstract

**Background:**

Nontuberculous mycobacteria are environmental mycobacteria that rarely cause human disease, especially in the central nervous system. Central nervous system infection by *Mycobacterium avium* complex, the most common pathogen among nontuberculous mycobacteria species, is rare and seldom reported, even in those with advanced human immunodeficiency virus infection. We describe a case of *Mycobacterium avium* complex meningitis with cerebral hemorrhage in an human immunodeficiency virus uninfected man in Vietnam.

**Case presentation:**

A 56-year-old Vietnamese man with hypertension was hospitalized with a 5-day history of headache, dizziness, low-grade fever, and unresponsive to 5 days of oral antibiotics. A brain magnetic resonance imaging, performed on day 12, showed hydrocephalus and lacunar infarct. The patient did not improve with 8 days of empirical treatment with ceftriaxone, vancomycin, dexamethasone, and meropenem, and was transferred to a referral hospital for tropical diseases. At the second hospital admission, a cerebrospinal fluid analysis showed a white cell count of 22,518 cells/μL with 81% neutrophils, protein 1.72 g/L, and glucose 0.85 mmol/L. Acid-fast bacilli smear of the cerebrospinal fluid was positive. Molecular testing of the cerebrospinal fluid was negative on GeneXpert Ultra testing, while the line probe assay was positive for *Mycobacterium avium*. Blood cultures at two sites, cerebrospinal fluid cultures for bacteria and fungi, and human immunodeficiency virus Ag/Ab test were negative. The patient was continuously administered meropenem with the addition of azithromycin, rifampin, and ethambutol. Then, 1 day after nontuberculous mycobacteria treatment, he developed right-sided hemiplegia, and brain computed tomography showed a hemorrhage in the parietal area, adjacent to the left lateral ventricle, and left lateral intraventricular hemorrhage shifts the midline to the right. He was transferred to the third referral general hospital and died 22 days after the onset of symptoms.

**Conclusion:**

Nontuberculous mycobacteria-central nervous system infection might mimic unresponsive pyogenic bacterial meningitis. A rapid and accurate diagnosis is essential for initiating appropriate therapy for this deadly disease.

## Background

Nontuberculous mycobacteria (NTM) are environmental mycobacteria commonly found in soil and water. Although rarely causing human disease, NTM, classified into rapid-growing (less than 7 days) and slow-growing (more than 7 days) groups, can lead to a broad spectrum of diseases, such as lung, lymphatic, skin and soft tissue, and central nervous system (CNS) infections [1, 2]. The rapid-growing mycobacteria have been increasingly acknowledged as emerging pathogens; however, they are not always life-threatening and can sometimes be effectively managed through appropriate source control measures alone [3]. The most prevalent and pathogenic NTM is the slow-growing, scotochromogenic *Mycobacterium avium* complex (MAC) [2].

*Mycobacterium avium *(*M*. *avium*) was the etiologic agent of more than 95% of opportunistic infections in immunocompromised patients, particularly in those with advanced human immunodeficiency virus (HIV) infection and acquired immunodeficiency syndrome (AIDS) [4]. *M*. *avium* was the most commonly isolated NTM species in South Korea [11,705 MAC isolates from 17,915 NTM respiratory specimens (65.3%)], Singapore [10 MAC isolates among 23 cases with persistent niacin-negative mycobacteria (43.5%)], Hong Kong [53 MAC isolated from sputum of 168 NTM pulmonary patients (31.5%)], and Taiwan [9204 NTM isolates from 283,394 clinical samples (30.0%)] [5]. However, *M*. *avium*-CNS infection is extremely rare, even in severely immune suppressed individuals [6].

Diagnosis of *M*. *avium* meningitis is challenging owing to its rarity and its nonspecific clinical and laboratory features and very few cases have been documented among immunocompetent patients [7–9]. With the rise in prevalence of NTM-associated diseases [5], whether NTM meningitis is extremely rare or neglected and understudied, hence underreported, is indistinguishable. We reported a persistent headache and neutrophilic meningitis complicated with cerebral hemorrhage in an HIV-negative Vietnamese adult man following an approach of clinical problem-solving.

## Case presentation

**Fig. 1:**
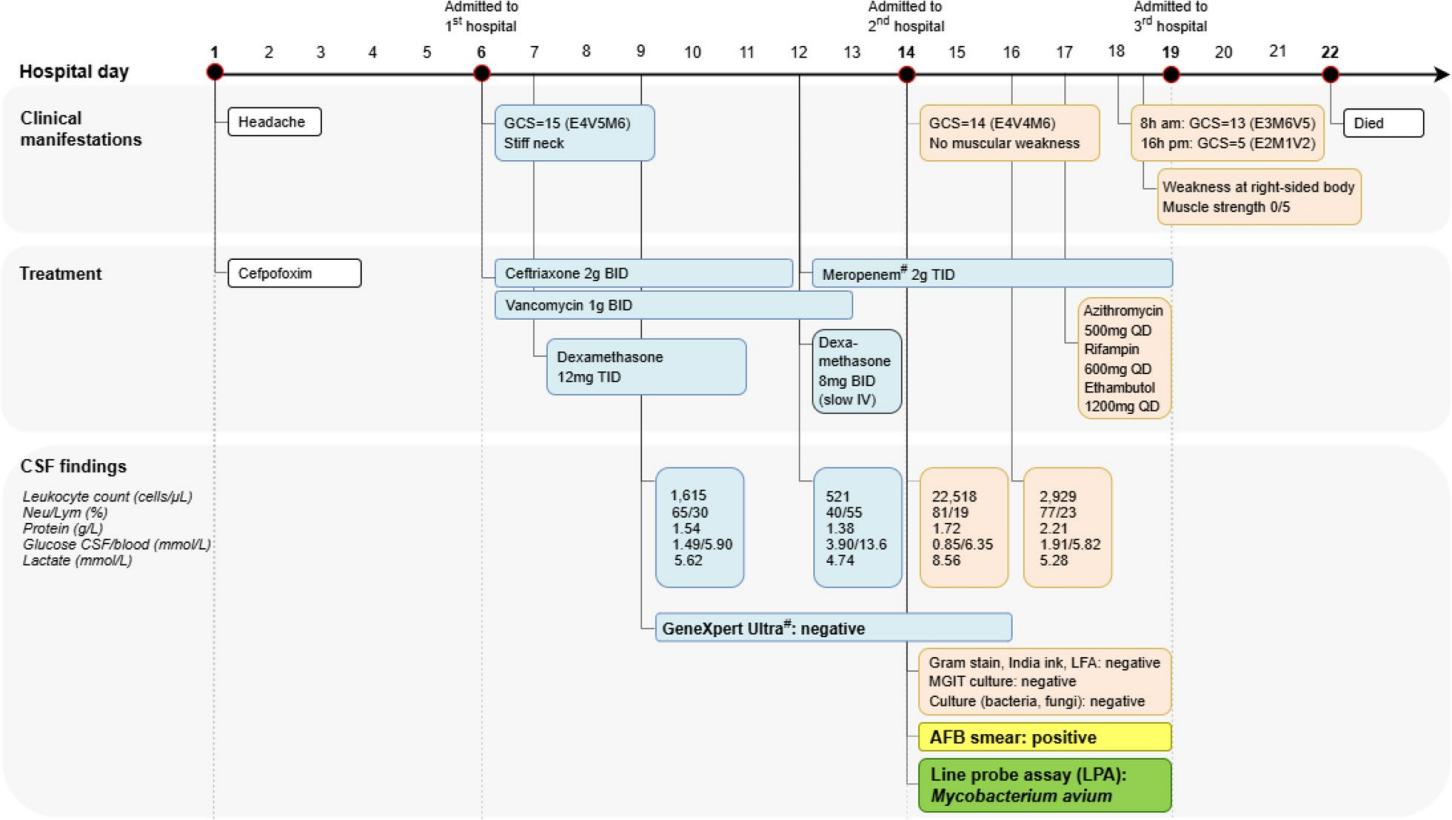
Summary of treatment and follow-up in a 56-year-old male with non-tuberculous mycobacteria meningitis. ^#^ at both first and second hospital, *AFB* acid-fast bacilli, *BID* twice a day, *CSF* cerebrospinal fluid, *GCS* Glasgow coma score, *IV* intravenous, *LPA* Line probe assay, *NTM* non-tuberculous mycobacteria, *PCR* polymerase chain reaction, *QD* once a day, *TID* three times a day, ( ) = hospitalization at the first tertiary hospital, () = treatment at the second tertiary hospital for tropical diseases

A hypertensive 56-year-old man visited a local clinic due to moderate to severe headache associated with low-grade fever, dizziness, and postprandial vomiting. The patient was otherwise healthy, but had been diagnosed with sinusitis and prescribed cefpodoxime for 5 days. His headache and other symptoms became more intense after 4 days of treatment, which led to hospitalization at a tertiary hospital in Ho Chi Minh City (HCMC), Vietnam at day 5 of illness (Fig. 1).

**Fig. 2:**
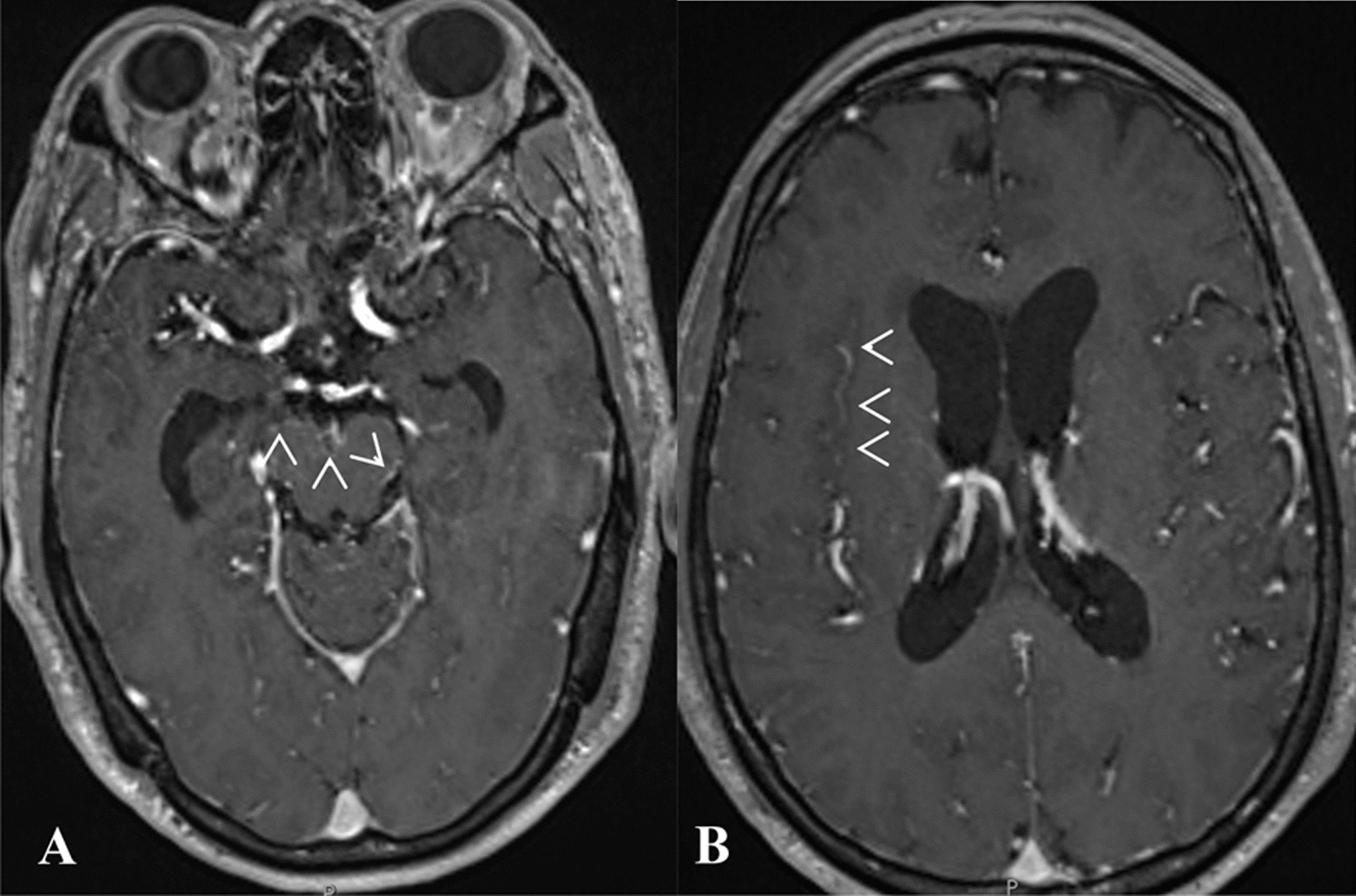
Hydrocephalus and leptomeningeal enhancement on magnetic resonance image on day 6 (re-analysis). Contrast enhanced axial T1-weighted images (**A**, **B**) reveal hydrocephalus with dilated frontal and temporal horns of lateral ventricles. Leptomeningeal enhancement noted (arrowheads) at basal cistern, ambient cistern (**A**) and the right Sylvian fissure (**B**)

On admission to the hospital, the patient had neck stiffness while was alert and orientated. No other neurological deficits was detected. An initial cranial magnetic resonance imaging (MRI) showed the maxillary sinus mucosa was thickened with no intracerebral hemorrhage (Fig. 2). His white-blood cell (WBC) count was 12.3 K/μL with 82% neutrophils, C-reaction protein was 12.3 mg/L level (reference range, < 5 mg/L) and pro-calcitonin was within the normal range (0.02 ng/mL). Lumbar puncture (LP) was performed, revealing cloudy cerebrospinal fluid (CSF), with 1615 cells/μL leukocytes (65% neutrophils, 30% lymphocytes) with other characteristics shown in Table 1 and Fig. 1. CSF GeneXpert Ultra (Cepheid, Sunnyvale, CA, USA) for *Mycobacterium tuberculosis* (*Mtb*) and Gram stain were negative. No parenchymal abnormality was detected on chest X-ray (CXR). He received ceftriaxone 2 g twice daily (BID) and vancomycin 1 g BID on the day of admission, followed by dexamethasone 12 mg three times daily (TID) from the next day.

**Table 1:** Summary of laboratory test results

Laboratory test results	Day of illness				
	6^a^	9^a^	14^b^	16^b^	19^b^
Complete blood count					
White blood cell (K/μL)	12.3	20	26.44	20.87	20.43
Neutrophils (%)	75	92.6	92.3	85.5	86.5
Lymphocytes (%)	–	–	1.9	5.5	4.2
Monocytes (%)	–	–	3.7	7.2	8
Eosinophils (%)	–	–	1.7	1.1	0.2
Red blood cell (M/μL)	–	–	4.17	4.41	4.31
Hemoglobin (g/dL)	–	–	13.3	14.1	13.3
Hematocrit (%)	–	–	37.9	42	41.4
Platelets (K/μL)	–	–	534	613	569
Electrolytes					
Sodium (mmol/L)	–	–	106	120	123
Potassium (mmol/L)	–	–	4.61	3.75	3.78
Chloride (mmol/L)	–	–	75	86	93
Creatinine (umol/L)	–	–	45	–	–
Glucose mmol/L	–	–	6.35	–	–
AST (GOT) (U/L)	–	–	16.6	–	17
ALT (GPT) (U/L)	–	–	28.5	–	28
HIV Ag/Ab combo	–	–	Negative	–	–
CSF parameters					
Turbidity	Cloudy	Mild cloudy	Mild cloudy	Cloudy	–
Leukocyte (cells/μL)	1615	521	22,518	2929	–
Red blood cell (cells/μL)	–	–	2000	0	–
Neutrophils (%)	65	40	81	77	–
Lymphocytes (%)	30	55	19	23	–
Total protein (g/L)	1.54	1.38	1.72	2.207	–
CSF glucose (mmol/L)	1.49	3.9	0.85	1.91	–
Blood glucose at the same time (mmol/L)	5.9	13.6	6.35	5.82	–
Lactate (mmol/L)	5.62	4.74	8.56	5.28	–

^a^At the first hospital

^b^At the second hospital

– Not available or not done, *CSF* cerebrospinal fluid, *MGIT* *Mycobacterium* growth indicator tube

**Fig. 3:**
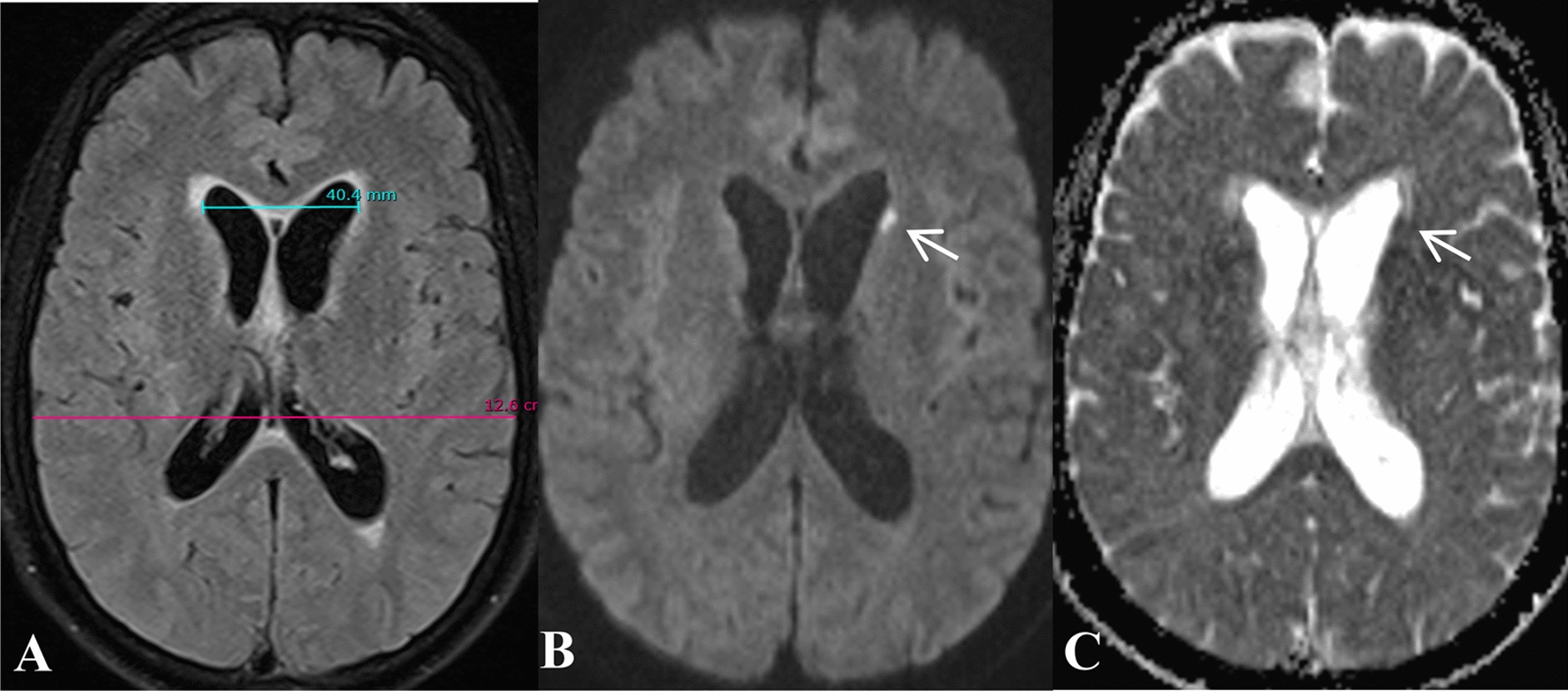
Hydrocephalus and lacunar infarct on magnetic resonance image on day 12 (re-analysis). Axial fluid-attenuated inversion recovery image (**A**) shows hydrocephalus with dilated frontal horns of lateral ventricles, Evans index 0.32. Axial Diffusion-weighted image (**B**) and ADC map (**C**) reveals recent lacunar infarct (arrows) with restricted diffusion at the left caudate nucleus. *NTM* nontuberculous mycobacteria

On day 9 of illness, WBC increased to 20.0 K/μL (92.6% neutrophils). A second LP was performed to reveal turbid fluid, with 521 cells/μL and lymphocyte predominance (Fig. 1). A second GeneXpert Ultra in CSF was negative, and a second CXR remained normal. Dexamethasone was stopped after 4 days of administration. Then, 3 days later, the patient’s cognitive status worsened, and WBC increased to 27.5 K/μL (83.7% neutrophils). Next, 2 g of meropenem TID was prescribed and dexamethasone was re-initiated at a decreased dose of 8 mg BID. The second cranial MRI revealed a hydrocephalus with dilated frontal horns of lateral ventricles and recent lacunar infarct with restricted diffusion at the left caudate nucleus (Fig. 3). The patient was transferred to our hospital, a tertiary referral hospital for infectious diseases, on day 14 of his illness.

The patient was a farmer and lived in the Mekong Delta region, approximately 50 km south of HCMC. Other than hypertension he had no significant comorbidities. He reported no use of analgesics, traditional medicines, corticosteroids, tobacco, alcohol, or cannabis, and had no history of head trauma or seizures. There was no significant family history of medical conditions. Upon admission to our hospital, the patient’s temperature was 37.5 °C (99.5 °F), blood pressure 160/90 mmHg, and the other vital signs were in normal range. Besides neck stiffness and suspected oral thrush, the physical examination was normal. The Glasgow coma score (GCS) was 14 (E4V4M6). He was mildly confused but otherwise his eyes were open and he executed the physician's directions with normal motor power. The laboratory tests showed leukocytosis (26.44 K/μL) with 92.3% neutrophils, hyponatremia (106 mmol/L, reference range, 135–145 mmol/L), and normal renal and hepatic function. Another LP was performed revealing cloudy CSF with 22,518 leukocytes/μL (81% neutrophils, 19% lymphocytes), 2000 red blood cells/μL, protein 1.72 g/L (reference range, 0–0.45 g/L), CSF:blood glucose of 0.85/6.35 mmol/L. CSF Gram and India ink stains, lateral flow assay for *Cryptococcus*, bacterial and fungal cultures were all negative (Table 1). The patient was treated with meropenem 2 g TID and hypertonic saline infusion but showed no clinical improvement.

Then, 2 days later, WBC decreased to 20.87 K/μL (85.5% neutrophils) and serum sodium increased to 120 mmol/L; however, no clinical improvement was observed. A fourth LP was performed to obtain CSF at 2929 leukocytes/uL (77% neutrophils, 23% lymphocytes), protein 2.21 g/L, CSF-to-serum glucose 1.91/5.82 mmol/L, and lactate 5.28 mmol/L (Table 1). CSF Ziehl Neelson smear revealed one acid-fast bacilli (AFB) per 100 fields but GeneXpert Ultra was negative. The Line Probe Assay (LPA) (Hain Lifescience GmbH, Nehren, Germany) detected *M*. *avium*. Serological tests for the HIV Ag/Ab combination were negative. Blood cultures were negative for both bacteria and fungi (Fig. 1). Treatment was initiated with azithromycin 500 mg once a day (QD), rifampin 600 mg QD, and ethambutol 1200 mg QD. No abnormalities were detected on CXRs.

**Fig. 4:**
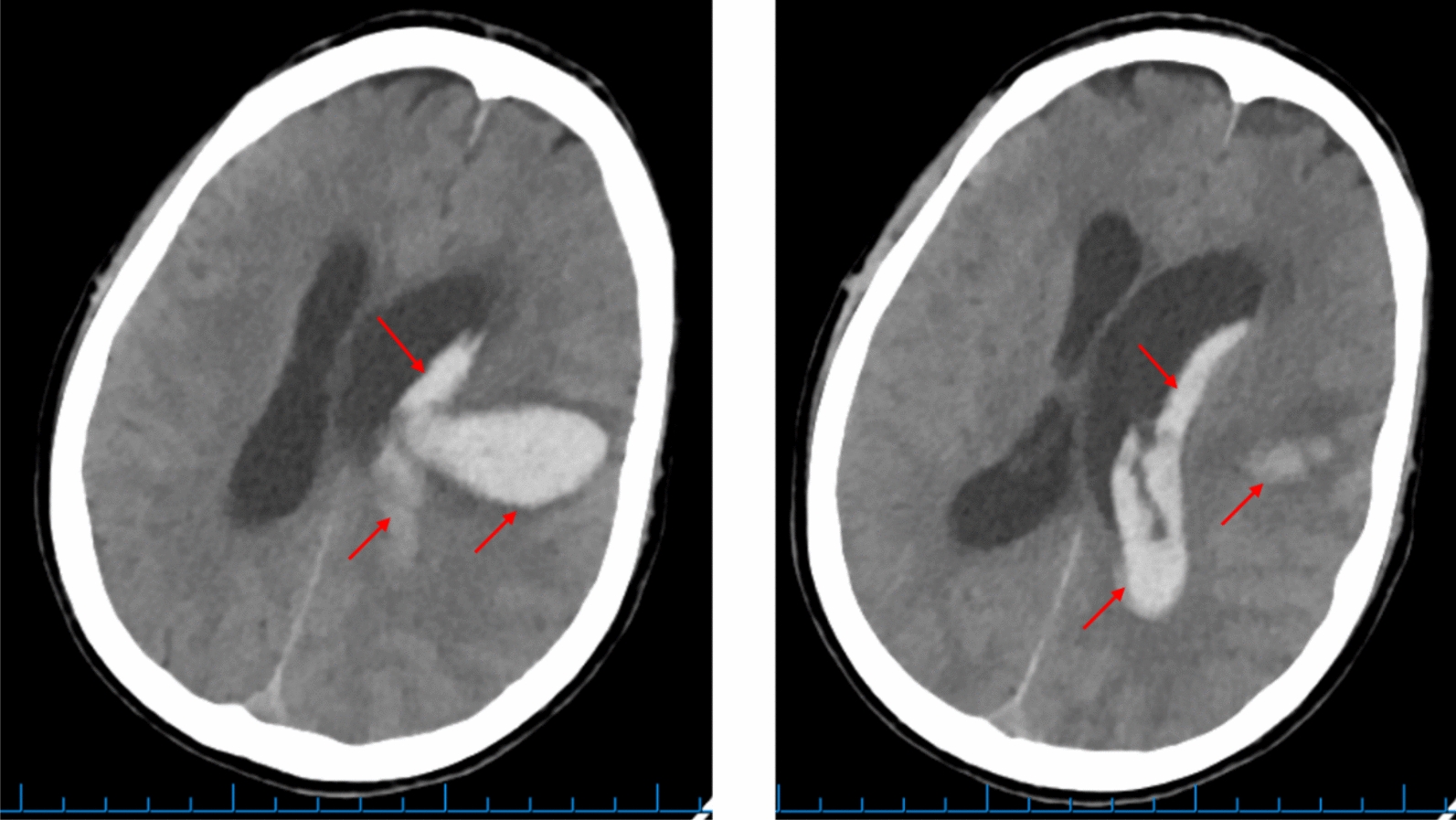
Hemorrhage on contrast-enhanced brain CT scan on day 19. Hemorrhage in the parietal area, adjacent to the left lateral ventricle (arrows), D# 2.9 × 4.3 × 4.7 cm, and left lateral intraventricular hemorrhage shifts the midline to the right, *CT scan* computed tomography scan

After 1 day of NTM treatment (day 18 of illness), the patient’s cognitive status suddenly deteriorated with GCS falling from 13 (E3M6V4) to five (E2M1V2). An emergency contrast-enhanced brain computed tomography scan revealed a hemorrhage in the parietal area, adjacent to the left lateral ventricle, and left lateral intraventricular hemorrhage shifts the midline to the right (Fig. 4). The patient was transferred to a specialist neurology/neurosurgical department of another nearby hospital. The diagnosis at transfer was left cerebral hemisphere hemorrhage, intraventricular hemorrhage associated with *M*. *avium* meningitis and a background of hypertension. Despite medical management, the patient died on day 22 of his illness.

## Discussion and conclusions

### Chief complaint approach and epidemiological problem-solving

Headache is a common clinical manifestation with various etiologies, which can be broadly classified into two categories: primary headache disorders (for example, migraine, tension-type headache, and cluster headache) and secondary headaches caused by extracranial (for example, sinusitis and glaucoma), intracranial (for example, post-trauma, hemorrhage, and infections), or systemic (for example, acute severe hypertension) disorders. Our patient received empirical antibiotics for the diagnosis of sinusitis although he did not have other signs of sinusitis, such as nasal obstruction, congestion, or rhinorrhea, besides headache. Moreover, he also experienced postprandial dizziness and vomiting, which favored other diagnoses, such as an intracranial disorder involving the CNS, systemic infections, or cranial vascular disorders (for example, subarachnoid hemorrhage). He resided in Vietnam, a low-middle-income country with a high burden of tuberculosis, where access to NTM diagnosis was only limited in tertiary hospitals. Furthermore, the lack of trained personnel in non-specialty hospitals to suspect and screen for NTM infections was a significant barrier to diagnosis. Other common community-acquired brain infections in this region include *Streptococcus suis*, *Angiostrongylus cantonensis* (the rat lung worm) causing eosinophilic meningitis, and Gram-negative bacterial meningitis associated with disseminated strongyloidiasis. Furthermore, the early-stage overlapping clinical and laboratory manifestations of central nervous system infections may contribute to delays in diagnosis. He did not report the use of immunosuppressive or glucocorticoid drugs, malignant comorbilities, and diabetes. Our patient was HIV-negative with no other history or features suggestive of immune compromise.

### Initial diagnostic and therapeutic problem-solving

The patient presented with signs and symptoms suggestive of meningitis, including neck stiffness, fever, headache, and vomiting. Bacterial meningitis was suspected due to neutrophilic leukocytosis in both serum and CSF, elevated serum C-reaction protein, increased CSF protein and lactate, and decreased CSF-to-serum glucose ratio. With an acute onset suggestive of bacterial meningitis, and unresponsiveness to previous oral antibiotics, treatment with broad spectrum intravenous antibiotics was promptly initiated. A third-generation cephalosporin, that is, 2 g of ceftriaxone BID, is the widely recommended first-line therapy for bacterial meningitis. Vancomycin was added because *Streptococcus pneumoniae* infection could not be ruled out. Close monitoring of his treatment response was necessary (with repeat LP) because tuberculous meningitis remained a possibility, even with negative GeneXpert Ultra. His condition deteriorated after 6 days of treatment with ceftriaxone, vancomycin, and dexamethasone. Therefore, 2 g of meropenem TID was added to cover multidrug-resistant Gram-negative organisms.

### Therapeutic response and comprehensively diagnostic problem-solving

Our patient had persistent and marked neutrophil leukocytosis in blood and CSF which suggested pyogenic bacterial meningitis, but they did not respond to empiric antibiotic therapy with meropenem and vancomycin. Although CSF characteristics still favored purulent bacterial meningitis, a CSF shifting from predominant neutrophils to lymphocytes warranted further evaluation for other potential etiologies such as tuberculosis. CSF bacterial and fungal stains and cultures were negative and HIV Ag/Ab was negative. The patient did not respond to broad-spectrum antibiotics, making the diagnosis of TBM more likely, although neutrophilic CSF findings were atypical for TBM. CSF GeneXpert Ultra were negative three times on day 6, 9, and 16 of illness, and AFB on day 16 were positive. This result led to suspect of NTM meningitis. LPA was performed to confirm the presence of *M*. *avium* in the CSF. All CSF mycobacterial cultures were negative. Diagnosis of NTM-CNS infection in this case was challenging because both clinical and laboratory features mimicked pyogenic bacterial meningitis. Severe hyponatremia (106 mmol/L) might be caused by cerebral salt wasting or the syndrome of inappropriate antidiuretic hormone secretion, which can be found in severe brain infection.

Prognostically, NTM meningitis has high mortality and requires prompt pathogen identification [10] to enable appropriate treatment. The gold standard for NTM diagnosis is culture on both liquid and solid media, which are time consuming (approximately 2 to 8 weeks) and might delay diagnosis. LPA provides a rapid species identification method for NTM and should be considered in GeneXpert Ultra negative and AFB positive in the CSF of meningitis patients. Treatment of NTM-CNS infection should be based on the susceptibility of the pathogen and the penetration ability of drugs through blood–brain barriers [11]. However, there is a lack of guideline for NTM-CNS infection due to its rarity. Combination of macrolides (azithromycin or clarithromycin), rifampin and ethambutol are usually recommended for pulmonary MAC infection treatment [11]. Among the three drugs, ethambutol has poor central nervous system penetrance [12, 13]. Therefore, to improve treatment outcome, other agents with good blood brain barrier penetrance and potent activities against both slowly and rapidly growing mycobacteria such as linezolid should be considered.

In this patient, the brain imaging found hydrocephalus and leptomeningeal enhancement on day 6 (Fig. 2), and worsening mental status and CSF findings prompted a repeat cranial MRI, which revealed hydrocephalus and lacunar infarct on day 12 of the illness (re-analyses), features suggestive of tuberculous meningitis (TBM) (Fig. 3**)**. Kwon *et al*. reported a wide diverse appearances and nonspecific radiologic features of NTM-CNS infection [14]. This disease is extremely rare, sporadically reported, and difficult to identify the causative bacteria. Meanwhile, the diagnosis of TBM, which is much more common, remains insensitive and delayed, leading to TB treatment decision for many cases without microbiological confirmation [10]. Therefore, NTM infection should be suspected among those whose clinical manifestations resemble TBM yet the CSF findings are atypical for TBM, especially those with poor treatment response.

Reviewing literature highlights disparities in clinical manifestations, comorbidities, diagnostic methods, neuroimaging findings, treatments, and outcomes in NTM-CNS infections [14–18]. A systematic review of 112 NTM-CNS cases (62.5% male) identified MAC as the most common etiology (35%), with a 37.5% case fatality rate. Male sex was found to be an independent risk factor for mortality, and neuroimaging frequently revealed intracranial abscesses (36.6%) and leptomeningeal enhancement (28%) [16]. Treatment regimens were determined on the basis of species identification, drug susceptibility, and expert opinion. Wang and colleagues reported a 55-year-old male with uncontrolled type 2 diabetes and an 8-day history of severe headache. *Mycobacterium avium* was identified by metagenomic next-generation sequencing (mNGS), and the patient was successfully treated with emperical antimycobacterial therapy and intravenous hydrocortisone [15]. In contrast, Kwon and colleagues reported a fatal case of NTM-CNS infection diagnosed by biopsy and polymerase chain reaction in a 30-year-old healthy male with a 6-month history of headaches. The patient died 1 month after brain biopsy despite anti-NTM treatment [14]. The clinical onset in our case, including fever, headache, neutrophilic pleocytosis, and leptomeningeal enhancement on MRI, was similar to prior reports. Despite variability in outcomes, NTM-CNS infections are severe and associated with a high mortality rate, underscoring the importance of early diagnosis and appropriate treatment.

In this middle-aged male, no known cause of immunosuppression was detected. However, autoantibodies were not performed to rule out autoimmune diseases. Recently, anticytokine autoantibodies (ACAAs) have been explored as an emerging mechanism of infection susceptibility for NTM, especially in healthy patients [8, 9, 19]. Disseminated nontuberculous mycobacterial and other opportunistic infections related to neutralizing anti-interferon-γ autoantibodies have been reported in East Asian adults without HIV [8, 20, 21]. Browne and colleagues observed a strong association between adult-onset immunodeficiency syndrome and high-titer neutralizing antibodies to interferon-γ detected in 88% of Asian adults, supporting the critical role of interferon-γ in controlling pathogens [7]. Many patients with anti–interferon-γ autoantibodies remain actively infected despite anti-microbial therapy. Browne *et al*. studied various patients from the USA, Taiwan, and Thailand infected with NTM meningitis and found that, unlike primary genetic immunodeficiencies that tend to present in childhood, ACAAs represent an emerging mechanism of infection susceptibility that is being increasingly recognized in previously healthy adults who develop severe opportunistic infections [7]. Although most reported cases were Asians, O’Connell *et al*. described a non-HIV Caucasian woman infected with MAC having high-titer IgG anti-interferon-γ autoantibodies (anti-IFN-γ autoantibodies) in plasma [9]. Cheng *et al*. showed that ACAA syndromes have diverse manifestations and neutralizing autoantibodies against specific cytokines, such as anti-IFN-γ autoantibodies in disseminated NTM, anti-granulocyte macrophage colony-stimulating factor autoantibodies in cryptococcal meningitis, and anti-type I IFN in severe COVID-19 [22].

In conclusion, NTM-CNS infection is rare and can mimic pyogenic bacterial meningitis. It should be suspected in those with unresponsive to broad antibiotic meningitis and screened by both culture and molecular tests. NTM-CNS infection in patients without HIV might associate with other causes of acquired immunodeficiency. Potential mechanisms, such as ACAAs, should be further studied to establish a multimodal treatment strategy for this deadly disease.

## Data Availability

Not applicable.

## Abbreviations

ACAAs: Anticytokine autoantibodies
AIDS: Acquired immunodeficiency syndrome
AFB: Acid-fast bacilli
BID: Twice daily
CNS: Central nervous system
CSF: Cerebrospinal fluid
CXR: Chest X-ray
GCS: Glasgow coma score
HCMC: Ho Chi Minh City
HIV: Human immunodeficiency virus
IFN: Interferon
LP: Lumbar puncture
LPA: Line Probe Assay
MAC: *Mycobacterium avium* Complex
MRI: Magnetic resonance imaging
*Mtb*: *Mycobacterium tuberculosis*
NTM: Nontuberculous mycobacteria
QD: Once a day
TBM: Tuberculous meningitis
TID: Three times daily
WBC: White-blood cell
